# Disparities in Prenatal Sexually Transmitted Infections among a Diverse Population of Foreign-Born and US-Born Women

**DOI:** 10.1007/s43032-022-00891-5

**Published:** 2022-02-25

**Authors:** Akaninyene Noah, Ashley V. Hill, Maria J. Perez-Patron, Abbey B. Berenson, Camilla R. Comeaux, Brandie D. Taylor

**Affiliations:** 1 Department of Obstetrics and Gynecology, Division of Basic Science and Translational Research, The University of Texas Medical Branch, MRB, 11.158A, 301 University Blvd, Galveston, TX USA; 2 Department of Epidemiology, Graduate School of Public Health, University of Pittsburgh, Pittsburgh, PA USA; 3 Department of Epidemiology and Biostatistics, School of Public Health, Texas A&M University, College Station, TX USA; 4 Center for Interdisciplinary Research in Women’s Health, Department of Obstetrics & Gynecology, The University of Texas Medical Branch, Galveston, TX USA; 5 Department of Preventive Medicine and Population Health, The University of Texas Medical Branch-Galveston, Galveston, TX USA

**Keywords:** Immigrant health, Sexually transmitted infections, Adverse pregnancy outcome, Health disparities

## Abstract

This study examined association between foreign-born (FB) status and a sexually transmitted infection (STI) diagnosis of *Chlamydia trachomatis*, *Neisseria gonorrhoeae*, or syphilis among a cohort of expecting mothers, and stratified by race/ethnicity. As a secondary analysis, subsequent adverse birth outcomes following STIs were examined. We used data from a large perinatal database to conduct a retrospective cohort study of 37,211 singleton births. Logistic regression was used to determine the association between FB status and STIs. We adjusted for maternal demographics, prior complications, and chronic disease. As a secondary analysis, we examined the association between STIs, and adverse birth outcomes stratified by FB status. FB women had lower odds of STI diagnosis (OR_adj_ 0.81, 95% CI 0.71–0.93); this was observed for each STI. Among Hispanic women, FB status did not reduce odds of STIs (OR_adj_ 0.89, 95% CI 0.76–1.04). However, FB Black women had reduced odds of STIs (OR_adj_ 0.53, 95% CI 0.36–0.79). Secondary analyses revealed that STIs increased odds of adverse birth outcomes among US-born Black women but not US-born Hispanic women. Among FB Black women, STIs increased odds of medically indicated preterm birth (OR_adj_ 3.77, 95% CI 1.19–12.00) and preeclampsia (OR_adj_ 2.35, 95% CI 1.02–5.42). This was not observed among FB Hispanic women. Previous studies suggest that FB women are less likely to have adverse birth outcomes; our study extends this observation to risk of prenatal STIs. However, FB status does not protect Black women against adverse birth outcomes following an STI.

## Background

Sexually transmitted infections (STI) have been increasing at record rates over the past several years [1], particularly common reportable STIs, *Chlamydia trachomatis*, *Neisseria gonorrhoeae*, and syphilis [1]. Recent data from 2019 shows a combined 2.5 million reported cases to the Centers for Disease Control (CDC) [1], a likely underestimation of the population burden given the asymptomatic natures of these pathogens. Determining effective strategies to reduce STI rates in the population has presented a significant challenge and racial disparities in STI prevalence persist [1]. In 2019, Hispanic and non-Hispanic (NH) Black women had 1.9 and 5 times higher reported chlamydia cases than NH Whites [1].

Understanding factors that influence STI risk and how that relates to birth outcomes in different populations is critical to optimizing screening strategies and clinical interventions. The extensive literature on the “healthy migrant effect” and the “Latina paradox” across multiple countries shows foreign-born (FB) status offering some protective effect against adverse pregnancy outcomes such as preterm birth and infant mortality [2–10]. Differences in STI prevalence have been observed by FB status [11]; however, few studies have examined the association between FB status and perinatal STIs, particularly for pregnant women. In addition, STIs have been linked to spontaneous abortion, preterm birth, and preeclampsia [12], yet there is limited literature on the risk of subsequent negative birth outcomes following an STI among FB and US-born mothers.

Our primary objective was to determine if there is an association between FB status and a perinatal STI diagnosis. We stratified this analysis by race/ethnicity. As a secondary analysis, we evaluated the role FB status plays on adverse birth outcomes and examined the association between STIs and adverse birth outcomes in FB and US-born women. We hypothesized that FB status may protect pregnant women against STIs and subsequent adverse pregnancy outcomes.

## Methods

### Study Design

We conducted a retrospective cohort study of 37,211 singleton livebirths using data from Peribank database and biorepository, which recruits women during admissions to labor and delivery [13]. First recorded births in Peribank were used for this analysis. Peribank collects data from electronic medical records, questionnaires, prenatal records, and in-person interviews. The Institutional Review Board at Texas Children’s Hospital and Baylor College of Medicine approved Peribank. All participants provided informed consent. For this analysis, the Institutional Review Board at the University of Texas Medical Branch determined that the research is non-human subjects research.

### Primary Exposure

**Fig. 1:**
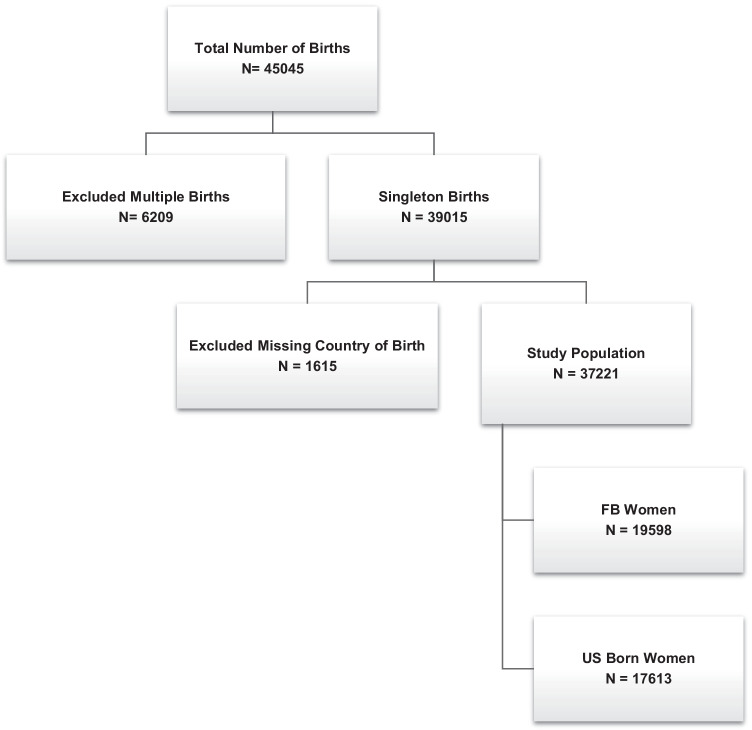
Flow chart of study population including exclusion criteria and composition. This figure shows the total number of births in the Peribank dataset between 2011 and 2020. After exclusions of multiple births, and missing country of birth, the total study population was 37,221

Our primary exposure was foreign-born status, using self-reported country of birth. Women with missing data on self-reported country of birth were excluded (4%) (Fig. 1). We did not observe any demographic or maternal characteristic differences between this group and women with a recorded country of birth.

### Primary Outcome

The primary study outcome was a composite variable composed of a positive diagnostic test for *Chlamydia trachomatis*, *Neisseria gonorrhoeae*, or syphilis. Nucleic acid amplification tests were used to diagnose *C. trachomatis* and *N. gonorrhoeae* per Centers for Disease Control and Prevention recommendations. Syphilis was diagnosed using a rapid plasma regain test [14]. Per hospital protocol, which follows recommendations of the American College of Obstetricians and Gynecologists (ACOG) [15], women were tested for each STI during the first prenatal visit and re-tested again in the third trimester. Both results were used in determining STI diagnosis. Treatment followed standard hospital protocols. A total of 33,936 (91.2%) women were screened for chlamydia, 33,796 (90.8%) for gonorrhea, and 36,422 (97.9%) for syphilis at the first prenatal visit. Hispanic women had the highest screening rates for chlamydia, gonorrhea, and syphilis at 92.5%, 91.9%, and 96.9% respectively, while non-Hispanic (NH) Blacks had the lowest screening rates for all three STIs at 85.2%, 85.5%, and 96.4% respectively.

### Secondary Analyses

As secondary analyses, we examined associations between foreign-born status and various pregnancy outcomes and between STI and pregnancy outcomes stratified by foreign-born status. Preterm birth was defined as a delivery occurring at less than 37 completed weeks of gestation and sub-grouped as medically indicated or spontaneous. Gestational age was measured using self-reported last menstrual period and updated with ultrasound estimation between 18 and 22 weeks’ gestation if necessary. Hypertensive disorders of pregnancy (HDP) included preeclampsia, superimposed preeclampsia, and gestational hypertension diagnosed by ACOG criteria [15]. Chorioamnionitis was clinically diagnosed as the presence of fever and intrauterine infection [16]. In addition, we pulled data indicating maternal ICU admission, stillbirth, placenta previa, placental abruption, and gestational diabetes.

### Covariates

Data was obtained on maternal age, body mass index, race/ethnicity, number of years lived in the USA, marital status, payment method, household income, and number of household members. Information on alcohol, tobacco, and drug use (heroin, methamphetamine, marijuana, cocaine) were obtained. Chronic health conditions included diabetes, cystic fibrosis, thyroid disease, and cancer. Data on infections was available (e.g., HIV, group B streptococcus, herpes simplex virus, bacterial vaginosis, cytomegalovirus). Pregnancy-related variables included infant sex, gestational age at first prenatal visit, gravidity, parity, and a history of abortion, gestational diabetes mellitus, preterm birth, and preeclampsia.

### Statistical Analysis

To compare demographics, behavioral characteristics, chronic health conditions, prior and current pregnancy characteristics between US-born and foreign-born women, we used a log-binomial regression model to calculate the point prevalence ratio (PR) and the 95% confidence intervals (CI). Directed acyclic graphs determined the final covariates included in the models. Multiple logistic regression was used to calculate odds ratios (OR) and 95% CIs for primary and secondary outcomes. The firth penalized approach was used for small sample sizes when appropriate. For the primary analyses, models were adjusted for race (except in race stratified models), age, education, marital status, payment method, substance use (drugs, alcohol, smoking), chronic health conditions, and prior adverse pregnancy outcomes. We conducted this analysis in the entire cohort, then stratified by race/ethnicity. Our secondary analysis model utilized the same covariates. We added other genital coinfections to the model but did not observe any difference in effect estimates, thus results are presented without this adjustment. The missingness of variables in the models ranged from 0.1 to 7%. Multiple imputation was used to account for missing covariate data [17].

We conducted several sensitivity analyses. First, we evaluated chlamydia, gonorrhea, and syphilis individually for all models and found few differences compared to the composite variable. We then conducted stratified analysis by race/ethnicity to determine if the magnitude and direction of our association varied across groups. We also considered if the number of years living in the USA modified our results. Lastly, to account for potential unmeasured confounding, we calculated an *E*-value score [18]. All analyses were done using SAS software version 9.4, SAS Institute, Cary, North Carolina.

## Results

### Population and Characteristics

Among women in our study, 19,598 (50.5%) were foreign-born, and 17,613 (49.5%) were US-born. The FB population was highly diverse with over 180 different nationalities represented: 80% of which came from Mexico and Spanish-speaking South/Central American countries. The remaining 20% originated from 52 different Asian and Middle Eastern countries (9%), 48 African countries (7%), 43 European countries (2%), and 18 north American and Caribbean countries (2%). US-born women were mostly NH White (41.2%) and Hispanic (31.8%), while FB women were largely Hispanic (81.2%), NH Black (7.2%), or other races (8.0%).

FB women (30, interquartile range [IQR] 25–34) and US-born women (29.0, IQR 25–34) had similar median age (Table 1). FB women were more likely to have a high school (PR 1.81, 95% CI 1.74–1.87) or less than high school education (PR 2.91, 95% CI 2.83–2.99), to use Medicaid/CHIP (PR 3.18 95% CI 3.07–3.30), or to have an unknown or no insurance payment method (PR 3.96, 95% CI 3.80–4.12). Also, FB women were more likely to be married (PR 1.11, 95% CI: 1.09–1.13) and have 5 + household members (PR: 1.46, 95% CI 1.41–1.50) compared to US-born women.

**Table 1:** Maternal characteristics and clinical variables among foreign-born and US-born women

*Demographics and clinical variables*	*Foreign born* *N* = *19,598*	*US born* *N* = *17,613*	*Prevalence ratio* *(95% CI)*
	***Maternal demographics***		
*Maternal age, median (IQR)*	30.0 (25.0–34.0)	29.0 (24.0–33.0)	1.0 (1.0–1.0)
*Race/ethnicity, n (%)*			
Non-Hispanic White	716 (3.7)	7253 (41.2)	Ref
Non-Hispanic Black	1402 (7.2)	4134 (23.5)	2.8 (2.6–3.1)
Hispanic	15,919 (81.2)	5604 (31.8)	8.2 (7.7–8.8)
Other	1558 (8.0)	620 (3.5)	8.0 (7.4–8.6)
*Missing*	5 (0.01)		
*Years living in the USA, n (%)*			
10 +	6831 (41.1)	13,168 (99.4)	Ref
6–10	3632 (21.8)	21 (0.2)	2.9 (2.9–3.0)
0–5	6173 (37.1)	63 (0.5)	2.9 (2.8–3.0)
*Missing*	7333 (19.7)		
*Education, n (%)*			
College degree and above	3675 (20.7)	8618 (51.0)	Ref
Some College	1116 (6.3)	3451 (20.4)	0.8 (0.8–0.9)
High School	4108 (23.2)	3507 (20.7)	1.8 (1.7–1.9)
Less than High School	8823 (49.8)	1330 (7.9)	2.9 (2.8–3.0)
*Missing*	2583 (6.9)		
*Method of payment, n (%)*			
Private	2435 (12.6)	9212 (53.4)	Ref
Medicaid/CHIP	15,241 (78.7)	7675 (44.5)	3.1 (1.0–3.2)
Other/unknown/none	1700 (8.8)	353 (2.1)	4.0 (3.8–4.1)
*Missing*	595 (1.6)		
*Household income, n (%)*			
$35,000 and above	2786 (18.6)	9707 (66.1)	Ref
Less than $35,000	12,219 (81.4)	4983 (33.9)	3.2 (3.1–3.3)
*Missing*	7516 (20.2)		
*Number of household members, n (%)*			
0–2	3084 (18.2)	5641 (33.3)	Ref
3–4	8754 (51.6)	8270 (48.8)	1.5 (1.4–1.5)
5 +	5130 (30.2)	3045 (18.0)	1.8 (1.7–1.8)
*Missing*	3287 (8.8)		
	***Current health indicators***		
*Body mass index (BMI), Median (IQR)*	31.1 (27.9–34.9)	31.2 (27.6–36.2)	1.0 (1.0–1.0)
*Missing*	2535 (6.8)		
^*a*^*Chronic health conditions, n (%)*			
No	18,334 (94.2)	16,230 (92.5)	Ref
Yes	1124 (5.8)	1325 (7.6)	0.9 (0.8–0.9)
*Missing*	198 (0.5)		
*Asthma, n (%)*			
No	18,901 (97.1)	15,639 (89.1)	Ref
Yes	557 (2.9)	1916 (10.9)	0.4 (0.4–0.4)
*Missing*	198 (0.5)		
*Mental health issues, n (%)*			
No	18,677 (96.0)	14,408 (82.1)	Ref
Yes	781 (4.0)	3147 (17.9)	0.4 (0.3–0.4)
*Missing*	198 (0.5)		
	***Behavioral characteristics***		
*Alcohol use, n (%)*			
No	15,622 (79.8)	5629 (32.0)	Ref
Yes	3946 (20.2)	11,969 (68.0)	0.3 (0.3–0.4)
*Missing*	45 (0.12)		
*Smoking, n (%)*			
No	18,555 (94.8)	14,043 (79.8)	Ref
Yes	1011 (5.2)	3558 (20.2)	0.4 (0.4–0.4)
*Missing*	44 (0.1)		
*Drug use, n (%)*			
*No*	19,290 (98.6)	15,350 (87.3)	Ref
*Yes*	275 (1.4)	2238 (12.7)	0.2 (0.2–0.2)
*Missing*	58 (0.2)		
	**Prior and current pregnancy complications**		
*Gravidity, n (%)*			
No prior pregnancy	4348 (22.2)	6247 (35.5)	Ref
1–2 prior pregnancies	9054 (46.3)	7952 (45.2)	1.3 (1.3–1.3)
2 + prior pregnancies	6188 (31.5)	3405 (19.3)	1.6 (1.5–1.6)
*Missing*	36 (0.1)		
*Prior GDMA, n (%)*			
*No*	18,341 (94.3)	17,260 (98.3)	Ref
*Yes*	1117 (5.7)	295 (1.7)	1.5 (1.5–1.6)
*Missing*	198 (0.5)		
*Prior abort, n (%)*			
*No*	14,079 (72.0)	12,137 (69.0)	Ref
*Yes*	5489 (28.1)	5460 (31.0)	0.9 (0.9–1.0)
*Missing*	46 (0.1)		
*Gestational age at 1st prenatal visit by trimester*			
*0–12 weeks*	9382 (55.1)	10,921 (68.0)	Ref
*13–26 weeks*	5985 (35.2)	4479 (27.9)	1.2 (1.2–1.3)
*27* + *weeks*	1659 (9.7)	654 (4.1)	1.6 (1.5–1.6)
*Missing*	4131 (11.1)		
*Maternal comorbidities, n (%)*			
*No*	10,019 (51.5)	13,046 (74.3)	Ref
*Yes*	9439 (48.5)	4509 (25.7)	1.6 (1.5–1.6)
*Missing*	198 (0.5)		
	***Infant characteristics***		
*Infant gender, n (%)*			
*Female*	9604 (49.1)	8552 (48.6)	Ref
*Male*	9976 (51.0)	9053 (51.4)	1.0 (1.0–1.0)
*Missing*	26 (0.07)		
*Admitted to NICU, n (%)*			
No	128 (97.0)	33 (51.6)	Ref
Yes	4 (3.0)	31 (48.4)	26.9 (9.2–78.4)
*Missing*	155 (0.4)		

^a^Chronic health conditions include autoimmune diseases, thyroid disease, chronic hypertension, cardiovascular disease, diabetes, endometriosis, gastrointestinal disorders (e.g., celiac disease). Prevalence ratios and 95% confidence intervals were calculated with log-binomial model

Across most behavioral and health characteristics, FB women were less likely to report using drugs (PR 0.20 95% CI 0.18–0.22), tobacco (PR 0.39 95% CI 0.37–0.41), and alcohol (PR 0.34 95% CI 0.33–0.35) during pregnancy. FB women were less likely to have asthma (PR 0.41 95% CI: 0.38–0.44), chronic health conditions (PR 0.87, 95% CI 0.83–0.90), and mental health conditions (PR 0.35, 95% CI 0.33–0.38), compared to US-born women. FB women were more likely to have gestational diabetes in a prior pregnancy (PR 1.54, 95% CI 1.49–1.58) and a first prenatal visit after 27 + gestational weeks (PR 1.55, 95% CI 1.53–1.59) compared to US-born women.

### Primary Analyses

Following adjustments, FB status was associated with lower odds of STI (OR_adj_ 0.81, 95% CI 0.71–0.93) (Table 2). Results were similar when we examined chlamydia (OR_adj_ 0.86, 95% CI 0.74–0.99), gonorrhea (OR_adj_ 0.52, 95% CI 0.34–0.80), and syphilis (OR_adj_ 0.66, 95% CI 0.47–0.93) separately. Among Hispanic women, there was a borderline association between FB status and STI (OR_adj_ 0.89, 95% CI 0.76–1.04). There was a significant association between FB status and gonorrhea (OR_adj_ 0.55, 95% CI 0.31–0.95) but not for syphilis (OR_adj_ 0.72, 95% CI 0.46–1.14) or chlamydia (OR_adj_ 0.93, 95% CI0.79–1.10).

**Table 2:** Association between foreign-born status and STIs among entire cohort

*STI*	*Foreign born* *n (%)* *N* = *19,598*	*US born* *n (%)* *N* = *17,613*	*OR*_*adj*_*, 95% CI*
*Any STI*	919 (4.7)	861 (4.9)	0.81 (0.71–0.93)
*Chlamydia*	796 (4.1)	700 (4.0)	0.86 (0.74–0.99)
*Gonorrhea*	59 (0.3)	99 (0.6)	0.52 (0.34–0.80)
*Syphilis*	113 (0.6)	147 (0.8)	0.66 (0.47–0.93)

^a^Adjusted for race, age, marital status, insurance, education, substance use, smoking, chronic health conditions, prior pregnancy complications

#### Stratification by Race/Ethnicity

The NH Black FB population had lower odds of STI (OR_adj_ 0.53, 95% CI 0.36–0.79). Results were similar for chlamydia (OR_adj_ 0.58, 95% CI 0.37–0.92) and syphilis (OR_adj_ 0.36, 95% CI 0.17–0.75), but we could not examine gonorrhea separately as there were only 2 cases (0.14%) among the NH Black FB population vs 79 (1.91%) cases among US-born Black women. There were only 5 (0.70%) FB NH White women with any STI compared to 106 (1.46%) US-born NH White women. Because of the low prevalence of STIs in this group, we could not examine stratified associations.

### Secondary Analyses

#### Foreign-Born Status and Adverse Birth Outcomes

**Fig. 2:**
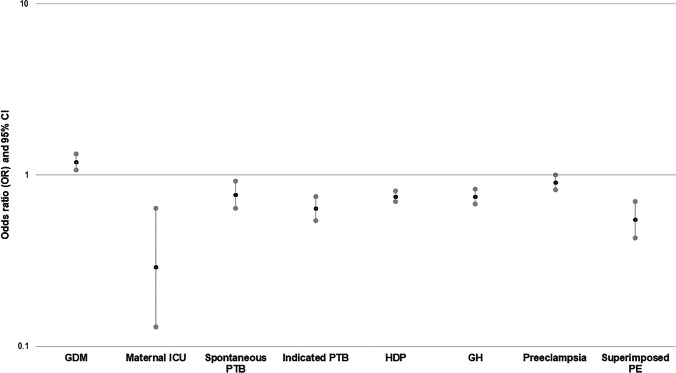
The association between FB status and adverse maternal outcomes. Results are adjusted for race, age, marital status, insurance, education, substance use, smoking, chronic health conditions, and pregnancy complications. Adjusted odds ratio (point estimate) of FB status and different adverse maternal outcomes are denoted by black dots; the grey dots represent the upper and lower limits of the 95% confidence interval. GDM, gestational diabetes Mellitus; ICU, intensive care unit; PTB, preterm birth; HDP, hypertensive disorders of pregnancy, GH, gestational hypertension; PE, preeclampsia

After adjustments, FB women appeared to have lower odds of maternal ICU admission (OR_adj_ 0.29, 95% CI 0.13–0.64), medically indicated PTB (OR_adj_ 0.64, 95% CI 0.54–0.75), and trends towards lower odds of spontaneous PTB (OR_adj_ 0.77, 95% CI 0.64–0.92) compared to US-born women (Fig. 2). Additionally, FB status was associated with lower odds of hypertensive disorders of pregnancy (OR_adj_ 0.75, 95% CI 0.70–0.81). When we examined each HDP individually, we observed a similar trend. In contrast, FB women experienced higher odds of gestational diabetes (OR_adj_ 1.19, 95% CI 1.07–1.33).

#### STIs and Adverse Birth Outcomes

There was no association between STIs and adverse pregnancy outcomes among the entire cohort of FB women. However, STI was associated with an increased odds of medically indicated PTB (OR_adj_ 3.77, 95% CI 1.19–12.00) and preeclampsia (OR_adj_ 2.35, 95% CI 1.02–5.42) among FB NH Black women. Among FB Hispanic women, STI was not associated with increased odds of adverse birth outcomes.

Among US-born women, STI was associated with stillbirth (OR_adj_ 2.93, 95% CI 1.09–7.88) and gestational hypertension (OR_adj_ 1.27, 95% CI 1.02–1.58). Among US-born NH Black women, STI was associated with increased odds of chorioamnionitis (OR_adj_ 1.68, 95% CI 1.05–2.71), placental abruption (OR_adj_ 3.09, 95% CI 1.25–7.65), and gestational hypertension (OR_adj_ 1.46, 95% 1.07–1.98). Among US-born Hispanic women, STI was not associated with an increased odds of adverse birth outcomes.

### Sensitivity Analysis

The years lived in the USA had little effect on outcomes. We did not find any evidence that living in the USA longer altered the association between FB status and STI diagnosis. Results were similar for adverse birth outcomes, except those FB women living in the USA 6–10 years (OR_adj_ 1.36, 95% CI 1.18–1.57) and 10 + years (OR_adj_ 1.34, 95% CI 1.19–1.52) did have higher odds of gestational diabetes compared to US-born women. There was no association between living in the USA 0–5 years and gestational diabetes (OR_adj_ 0.96, 95% CI 0.84–1.10).

## Discussion

Our findings suggest that FB women may have a lower odds of prenatal chlamydia, gonorrhea, and syphilis than US-born women. After stratifying by race/ethnicity, these associations were only observed among the NH Black FB population for all three STIs. Hispanic FB women had lower odds of gonorrhea only. STIs were associated with adverse pregnancy outcomes among the NH Black FB and US-born populations. Hispanic women with an STI did not have higher odds of adverse pregnancy outcomes.

Negative acculturation theory [19] suggests that longer time in the USA leads to increases in unhealthy behaviors. However, we did not find that length of time in the USA affected the association between FB status and STIs. A variety of reasons could explain reduced odds of STIs among FB women, one being the disparities in the onset of prenatal care among FB and US-born women. US-born women were more likely to present for prenatal care earlier (within 12 weeks). While FB women were more likely to present for prenatal care in the third trimester, there is a possibility of underdiagnosed STIs, if STIs are resolved prior to initiation of care or had been treated elsewhere and not recorded in the medical records. However, we observed higher STI screening rates among FB women.

Another reason could be cultural differences and norms, leading to a buffering effect among our FB study population, which may reduce risky behavior associated with higher STI rates. Cultural-based protective factors such as family, community connectedness and a strong sense of ethnic identity and norms have been shown to mitigate risky and self-harmful behaviors among migrants [20, 21]. The FB population had significantly lower rates of drug, tobacco, and alcohol use, compared to US-born women. Lastly, leading theories explaining this paradox have also included a selection bias where mostly healthier and more mobile people migrate to a different country [7, 19]. We did observe lower rates of chronic health conditions among our FB population.

Aligned with other research, FB women primarily had lower odds of most adverse perinatal outcomes [3, 5, 7, 9], apart from gestational diabetes [22]. A prior investigation among immigrants in New York also showed a higher risk of developing gestational diabetes than US-born women [22]. This could be due to the high stress exposure that many migrants often face, cultural differences in diet, and the high prevalence and accessibility of sugary food in the US diet, which could be used as coping mechanisms [23]. Also, we did observe that living in the USA longer increased GDM risk among FB women, further supporting this theory.

Overall, FB women with an STI did not have increased odds of adverse pregnancy outcomes, despite higher rates of late prenatal care. In contrast, US-born women with an STI had increased odds of hypertensive disorders and stillbirth. Interestingly, race/ethnicity modified this association. FB Black women with an STI were more likely to have a medically indicated preterm birth and preeclampsia. US-Born Black women with an STI had increased odds of chorioamnionitis, gestational hypertension, and placental abruption. This was not observed in Hispanic women. It is generally accepted that STIs result in poor birth outcomes. Still, associations between STIs and these specific outcomes have been inconsistent due to variations in study design, data quality, and populations examined [21–23]. These studies did not examine FB status nor stratify by race/ethnicity. One possible reason for our results among FB Black women could be due to the accumulative effects of stress due to adjusting to a new social culture as well as language barriers and racism that many immigrants to the USA encounter. Studies have shown that moderate-to-high stress levels can play an erosive role in health, and perceived racism can lead to worse birth outcomes among expectant mothers [24, 25]. Thus, many immigrants’ high levels of stress could make them more susceptible to adverse birth outcomes. This may be more difficult for NH Blacks than Hispanics, who have been shown to have greater resilience [26]. Among diverse populations, maternal nativity and race/ethnicity might inform prenatal STI clinical management. Efforts are needed to ensure that Black women receive prompt screening, treatment, and expedited partner therapy to reduce the risk of subsequent adverse pregnancy outcomes. However, more research is needed before specific clinical recommendations can be provided.

Failure to account for social determinants of health and population characteristics in research can miss important historical context and risk factors and result in ineffective policy, preventative, and clinical management recommendations [27]. Research should explore the specific risk factors that increase STI-associated adverse outcomes in Black women and/or factors that might protect STI-infected Hispanic women. In our study, FB women were more likely to identify as racial/ethnic minorities, have less time living in the USA, have a high school education or less, were lower income, and had more people in their household. It is likely that FB pregnant women in the USA have more unaddressed social determinants of health compared to US-born women that influence their health outcomes for a myriad of reasons [28, 29]. Although not the focus of this investigation, exploring the context of pregnant FB women’s lived experiences during transition to the USA and after, in relation to their STI risk will be helpful to tailor strategies that address the specific needs of FB perinatal populations. Biological mechanisms linking STIs to specific birth outcomes also need exploration as this is a significant gap in the literature. Identifying factors that drive the lower prevalence of STIs among FB women would also be important to update screening recommendations. Research along these lines may assist in developing culturally appropriate interventions to reduce STI risk before pregnancy. Investigations should move beyond the inclusion of race/ethnicity as a covariate and consider complex relationships between FB status and race/ethnicity in identifying risks for specific birth outcomes following an STI.

Our study has several strengths, including a large, diverse study population with over 180 different nationalities being represented. Over 90% of study participants were tested for an STI. Those not tested were more likely to be under 25, but age is not associated with FB status in this population. We did observe differences in screening rates by race/ethnicity where Hispanic women were more likely to be screened. Those included could reasonably be more likely to be foreign-born and slightly less likely to have an STI, which could bias our results. There is the possibility of a self-selection bias as migrants with uncertain visa/legal status may not consent to be part of the study data collection of personal information. However, Peribank represents ~ 85% of births across their clinics. Most of the literature on this subject matter uses birth record data, which has a high amount of missing data, especially on sensitive issues such as STI status. Missingness was low in our population except for a few variables that were not included in multivariable models. Multiple imputation was used in our study to address missing covariate data. Although we have some information about mental health, we were unable to measure high stress and discrimination. The presence of moderate unmeasured confounding could bias results. To determine this, we calculated *E*-value scores [18]. The lowest *E*-value was 1.60 (1.11 for the confidence interval closest to the null) for the association between FB status and chlamydia; thus, the confounder would need to have an effect of 1.60 or higher with both exposure and outcome to bias that association. Multiple comparisons are suggested to lead to an increase in type I error, but correction enhances type II error [30]. We followed the recommendations by Rothman and others [31] and focused our study on reporting effect estimates and confidence intervals, rather than *p*-values, and interpreting our results with caution and consideration of potential bias.

Our study contributes to the literature by showing that FB women may have a reduced risk of perinatal STIs, particularly NH Black women. However, once an STI is contracted, FB Black women may have increased odds of indicated preterm birth and preeclampsia. This may suggest that efforts to increase adequate health care among FB women are warranted. We cannot ignore the finding that NH Black women who contracted an STI had increased odds of several adverse birth outcomes, not observed in other racial/ethnic groups. Future research should explore if the accumulative impact of discrimination and racism is a key force for the increased risk of STIs in US-born vs. FB Black women.

## Data Availability

The data underlying this article were provided by Peribank under license/by permission. Access to data requires permission from Peribank [13].

## Code availability

All analyses were done using SAS software version 9.4, SAS Institute, Cary, North Carolina. Access to code is available upon request.
